# Supplementary material for: Formation Thermodynamics of Carbamazepine with Benzamide, Para-Hydroxybenzamide and Isonicotinamide Cocrystals: Experimental and Theoretical Study
Source: Pharmaceutics. 2022 Sep 6;14(9):1881. doi: 10.3390/pharmaceutics14091881 (PMC9501737; doi:10.3390/pharmaceutics14091881)
Supplement: Supplementary file 1 [file pharmaceutics-14-01881-s001.zip › pharmaceutics-1888368-supplementary.pdf]

pharmaceutics

Supplementary Materials: Formation Thermodynamics of Carbamazepine with Benzamide, *para*–Hydroxybenzamide and Isonicotinamide Cocrystals: Experimental and Theoretical Study

Alex N. Manin, Denis E. Boycov, Olga R. Simonova, Tatyana V. Volkova, Andrei V. Churakov and German L. Perlovich

Table S1. Experimental values of solubility, solubility product (K_{sp}) and free energy of formation (ΔG_f^0) of CBZ, coformers and cocrystals [CBZ + BZA], [CBZ + 4–OH–BZA], and [CBZ + INAM] in acetonitrile at 293.15 K, 298.15 K, 303.15 K, 308.15 K and 313.15 K.

		Temperature, K				
		293.15	298.15	303.15	308.15	313.15
CBZ	S^p_{CBZ}·10¹, mol·L⁻¹	1.48 ± 0.04	1.68 ± 0.05	1.95 ± 0.05	2.21 ± 0.06	2.45 ± 0.06
BZA	S^p_{BZA}·10¹, mol·L⁻¹	3.92 ± 0.03	4.94 ± 0.11	6.19 ± 0.14	7.79 ± 0.15	10.20 ± 0.17
4–OH–BZA	$S^p_{4-OH-BZA}$·10¹, mol·L⁻¹	1.10 ± 0.02	1.26 ± 0.03	1.45 ± 0.04	1.69 ± 0.05	1.92 ± 0.05
INAM	S^p_{INAM}·10¹, mol·L⁻¹	1.02 ± 0.02	1.27 ± 0.03	1.43 ± 0.03	1.80 ± 0.03	2.06 ± 0.04
[CBZ+BZA]	S_{CC}^{exp} ·10¹, mol·L⁻¹	0.99 ± 0.05	1.10 ± 0.06	1.20 ± 0.05	1.36 ± 0.05	1.45 ± 0.06
	K_{sp} ·10², (mol·L⁻¹)²	0.97 ± 0.05	1.22 ± 0.05	1.45 ± 0.05	1.85 ± 0.06	2.11 ± 0.07
	ΔG_f^0, kJ·mol⁻¹	−4.36 ± 0.33	−4.75 ± 0.34	−5.34 ± 0.42	−5.72 ± 0.45	−6.44 ± 0.48
[CBZ+4–OH–BZA]	S_{CC}^{exp} ·10², mol·L⁻¹	3.12 ± 0.06	3.77 ± 0.08	4.46 ± 0.11	5.34 ± 0.13	5.89 ± 0.18
	K_{sp} ·10³, (mol·L⁻¹)²	0.97 ± 0.04	1.42 ± 0.05	1.99 ± 0.10	2.85 ± 0.10	3.47 ± 0.12
	ΔG_f^0, kJ·mol⁻¹	−6.87 ± 0.48	−6.69 ± 0.45	−6.68 ± 0.42	−6.58 ± 0.40	−6.45 ± 0.36
[CBZ+INAM]	S_{CC}^{exp} ·10¹, mol·L⁻¹	0.86 ± 0.06	0.95 ± 0.04	1.04 ± 0.03	1.12 ± 0.03	1.23 ± 0.04
	K_{sp} ·10², (mol·L⁻¹)²	0.74 ± 0.03	0.90 ± 0.04	1.08 ± 0.05	1.25 ± 0.05	1.50 ± 0.07
	ΔG_f^0, kJ·mol⁻¹	−1.74 ± 0.13	−2.13 ± 0.14	−2.40 ± 0.16	−2.98 ± 0.16	−3.16 ± 0.21

Table S2. Experimental values of [CBZ + CF] (1:1) cocrystal solubility product (K_{sp} in (mol·L^{-1})^2), intrinsic solubility ($S^0(CBZ)$ in mol·L^{-1}) of CBZ and ($S^0(CF)$ in mol·L^{-1}) coformer and Gibbs energy of cocrystal formation reaction (in kJ·mol^{-1}).

N	Coformer	Solvent	$\log(K_{sp})$	$\log(S^0(CBZ))$	$\log(S^0(CF))$	ΔG_f^0	Ref[a]
1	Cinnamic Acid	Water	−5.9	−3.2	−2.46	−1.42	[1]
2	Emodin	Ethanol	−4.28	−0.97	−1.74	−8.91	[2]
3	Emodin	2−propanol	−4.82	−1.36	−1.68	−10.21	[2]
4	Emodin	Ethyl acetate	−4.64	−1.32	−1.43	−10.77	[2]
5	Emodin	Acetone	−4.07	−1.27	−1.29	−8.58	[2]
6	Nicotinamide	Ethyl acetate	−3.17	−1.31	−0.96	−5.17	[3]
7	Saccharin	2−propanol	−3.41	−1.3	−0.8	−7.5	[3]
8	Saccharin	Ethyl acetate	−2.6	−1.31	−0.74	−3.13	[3]
9	Saccharin	Ethanol	−2.38	−0.85	−0.62	−5.15	[3]
10	Saccharin	Methanol	−2.22	−0.4	−0.46	−4.6	[4]
11	Nicotinamide	2−propanol	−2.43	−1.3	−0.2	−5.31	[3]
12	Paeonol	2−propanol	−2.32	−1.36	−0.12	−4.83	[2]
13	Glutaric acid	Ethyl acetate	−2.05	−1.31	0	−4.23	[3]
14	Nicotinamide	Ethanol	−1.66	−0.85	0.04	−4.82	[3]
15	Paeonol	Ethanol	−1.74	−0.97	0.08	−4.84	[2]
16	Paeonol	Methanol	−0.84	−0.49	0.22	−3.19	[2]
17	Glutaric acid	Ethanol	−1.03	−0.85	0.45	−3.54	[3]
18	Glutaric acid	2−propanol	−1.31	−1.3	0.56	−3.23	[3]

Table S3. Some characteristics of cocrystals studied for estimation of formation thermodynamics [a].

N	API	Coformer (CF)	Stoich	$T_m(API)$ [°C]	$T_m(CF)$ [°C]	$T_m(CC)$ [°C]	$\Delta G_{sub}^{0,298}(API)$ [kJ·mol^{-1}]	$\Delta H_{sub}^{0,298}(API)$ [kJ·mol^{-1}]	$\Delta G_{sub}^{0,298}(CF)$ [kJ·mol^{-1}]	$\Delta H_{sub}^{0,298}(CF)$ [kJ·mol^{-1}]
1	CBZ	BZA	1:1	156.0	127.0	155.3	58.5	114.20	41.6	96.9
2	CBZ	4-OH-BZA	1:1	156.0	159.9	172.2	58.5	114.20	58.9	117.8
3	CBZ	INAM	1:1	156.0	161.0	154.8	58.5	114.20	49.1	117.0
19	CBZ	Saccharin	1:1	156.0	227.9	176.0	58.5	114.20	60.5	130.2

[a] $T_m(API)$, $T_m(CF)$, $T_m(CC)$ are the melting temperatures of the Active Pharmaceutical Ingredient, Coformer and Cocrystal, respectively; $\Delta G_{sub}^{0,298}(API)$, $\Delta H_{sub}^{0,298}(API)$, $\Delta G_{sub}^{0,298}(CF)$, $\Delta H_{sub}^{0,298}(CF)$ are the sublimation Gibbs energies and enthalpies of the Active Pharmaceutical Ingredient and Coformer.

Table S4. Summary of the H-bond propensity calculation for the cocrystals, including types (A:A, A:B or B:B) of the bonds. The final column indicates if the interaction listed is observed in the crystal structure.

[CBZ+BZA] (1:1)					
Interaction	Donor	Acceptor	Propensity	Type	
1	N26	O1	0.83	B:A	+
2	N26	O27	0.75	B:B	+
3	N18	O1	0.65	A:A	+
4	N18	O27	0.54	A:B	+

Functional group definitions for the CBZ+BZA cocrystal system. The functional groups defined are carbamoyl_2 (**N26** and **O27**) and T3NH0_amide (**N18** and **O1**)

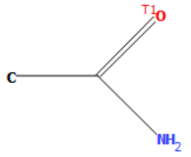

carbamoyl_2

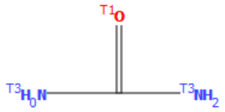

T3NH0_amide

[CBZ+4-OHBZA] (1:1)					
Interaction	Donor	Acceptor	Propensity	Type	
1	N26	O1	0.80	B:A	
2	N26	O27	0.73	B:B	+
3	N18	O1	0.60	A:A	+
4	O28	O1	0.53	B:A	+
5	N18	O27	0.52	A:B	
6	N26	O28	0.47	B:B	
7	O28	O27	0.44	B:B	
8	N18	O28	0.26	A:B	+
9	O28	O28	0.21	B:B	

Functional group definitions for the [CBZ+4-OHBZA] cocrystal system. The functional groups defined are carbamoyl_2 (**N26** and **O27**), ar_oh (**O28**) and T3NH0_amide (**N18** and **O1**)

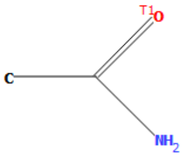

carbamoyl_2

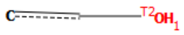

ar_oh

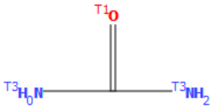

T3NH0_amide

[CBZ+INAM] (1:1)					
Interaction	Donor	Acceptor	Propensity	Type	
1	N27	N22	0.79	B:B	
2	N27	O1	0.74	B:A	+
3	N18	N22	0.71	A:B	
4	N27	O26	0.68	B:B	+
5	N18	O1	0.64	A:A	+
6	N18	O26	0.57	A:B	+

Functional group definitions for the [CBZ+INAM] cocrystal system. The functional groups defined are carbamoyl_2 (**N27** and **O26**), aromatic_nitrogen (**N22**) and T3NH0_amide (**N18** and **O1**)

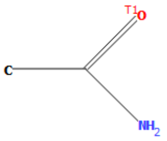

carbamoyl_2

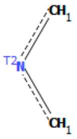

aromatic_nitrogen

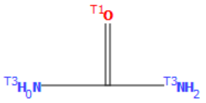

T3NH0_amide

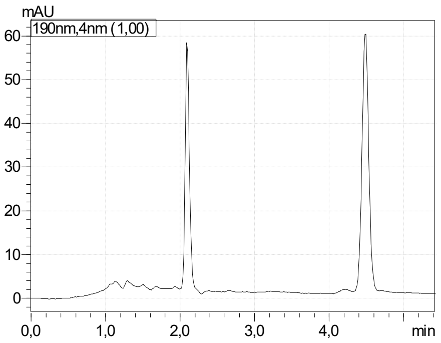

(**a**)

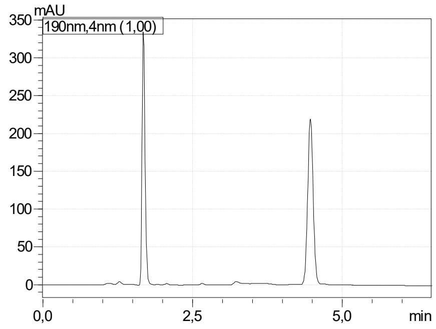

(**b**)

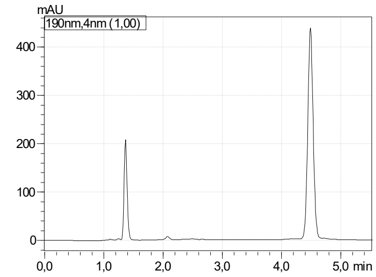

(**c**)

Figure S1 Chromatograms of cocrystals obtained at 298.15 K. (**a**) [CBZ + BZA], (**b**) [CBZ + 4-OH-BZA], (**c**) [CBZ + INAM].

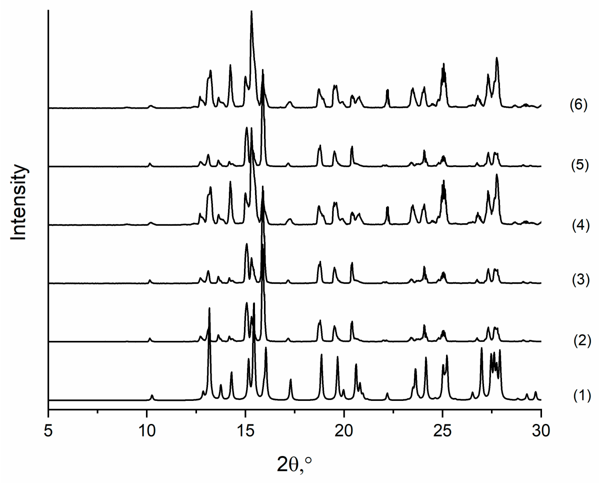
Intensity
2θ,°
5
10
15
20
25
30
(1)
(2)
(3)
(4)
(5)
(6)

(**a**)

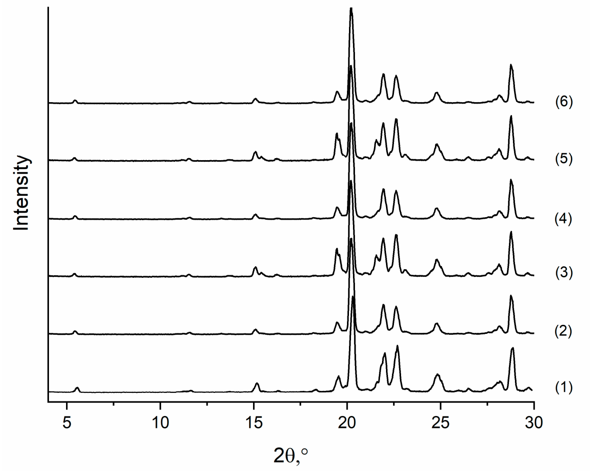
Intensity
2θ,°
5
10
15
20
25
30
(1)
(2)
(3)
(4)
(5)
(6)

(**b**)

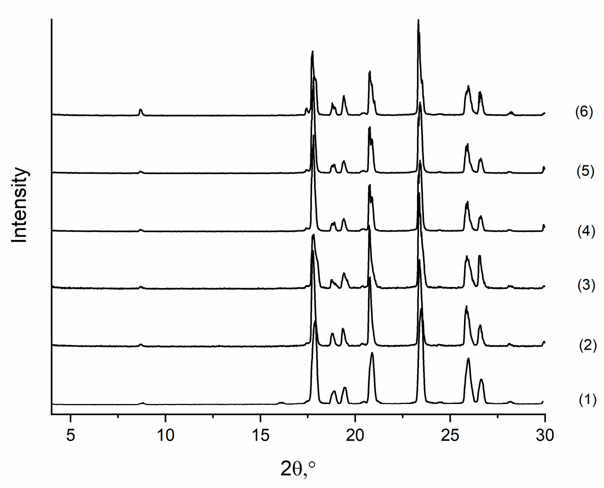
Intensity
2θ,°
5
10
15
20
25
30
(1)
(2)
(3)
(4)
(5)
(6)

(**c**)

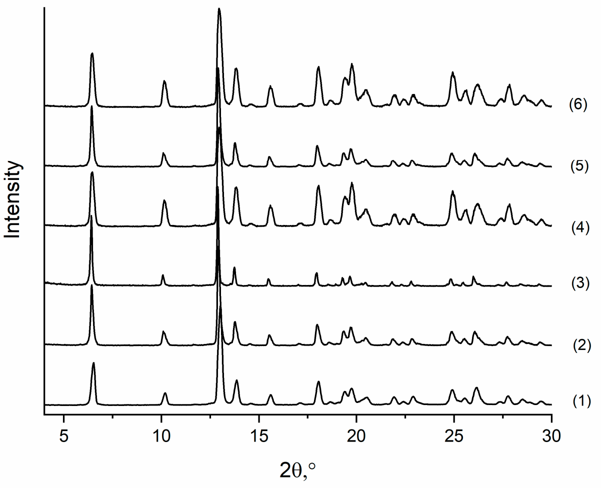
Intensity
2θ,°
5
10
15
20
25
30
(1)
(2)
(3)
(4)
(5)
(6)

(**d**)

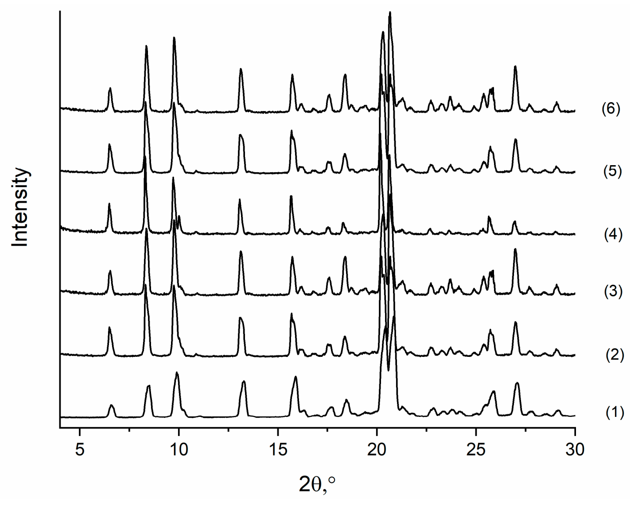
Intensity
2θ,°
5
10
15
20
25
30
(1)
(2)
(3)
(4)
(5)
(6)

(**e**)

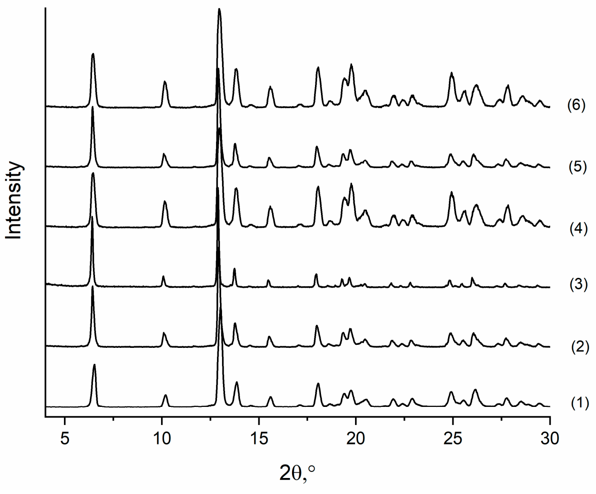
Intensity
2θ,°
5
10
15
20
25
30
(1)
(2)
(3)
(4)
(5)
(6)

(**f**)

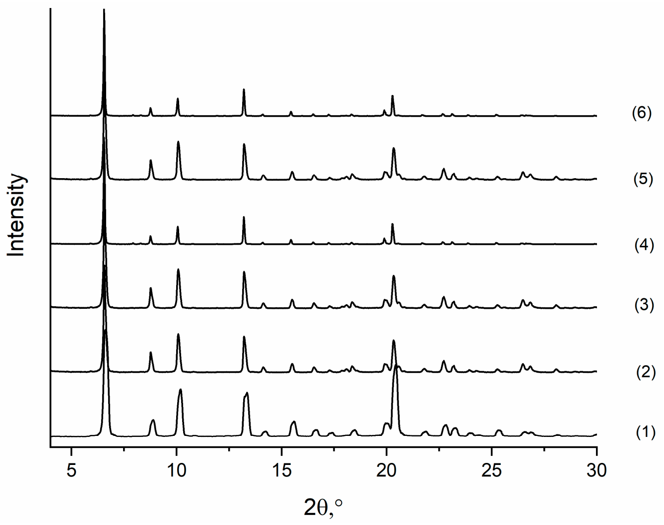

(g)

Figure S2. Comparison of experimental PXRD patterns of initial components and cocrystals with phases after experiment: (**a**) CBZ, (**b**) 4-OH-BZA, (**c**) INAM, (**d**) BZA, (**e**) [CBZ+4-OH-BZA] (1:1), (**f**) [CBZ+INAM] (1:1), (**g**) [CBZ+BZA] (1:1). (1) Initial phase, (2) 20 °C, (3) 25 °C, (4) 30 °C, (5) 35 °C, (6) 40 °C.

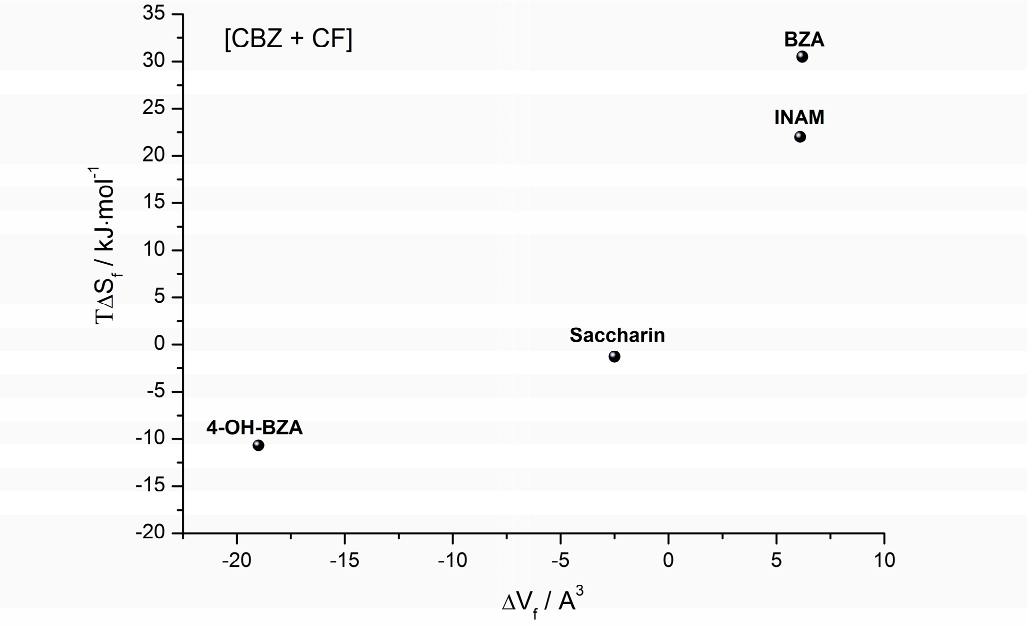

Figure S3. Dependence of $T\Delta S_f^{298}(\exp)$ versus $\Delta V_f(CC)$ for cocrystals [CBZ + CF].

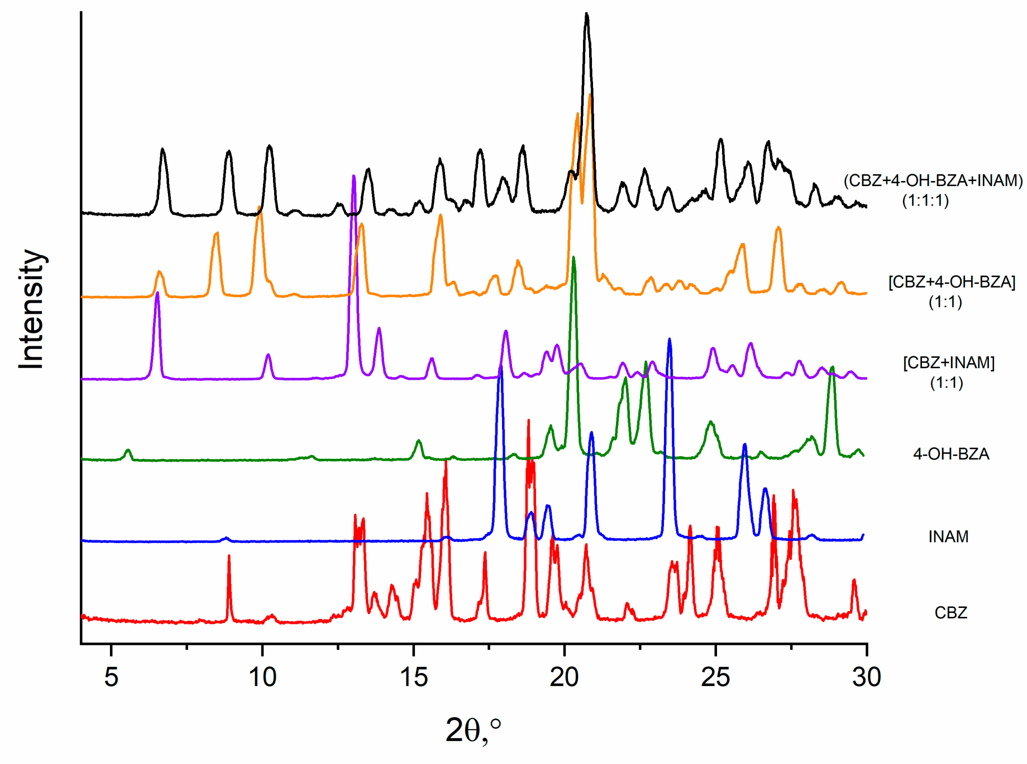

Figure S4. Comparison of the PXRD patterns: CBZ (red), INAM (blue), 4-OH-BZA (green), [CBZ+INAM] (1:1) (violet), [CBZ+4-OH-BZA] (1:1) (orange) and (CBZ+4-OH-BZA+INAM) (1:1:1) (black).

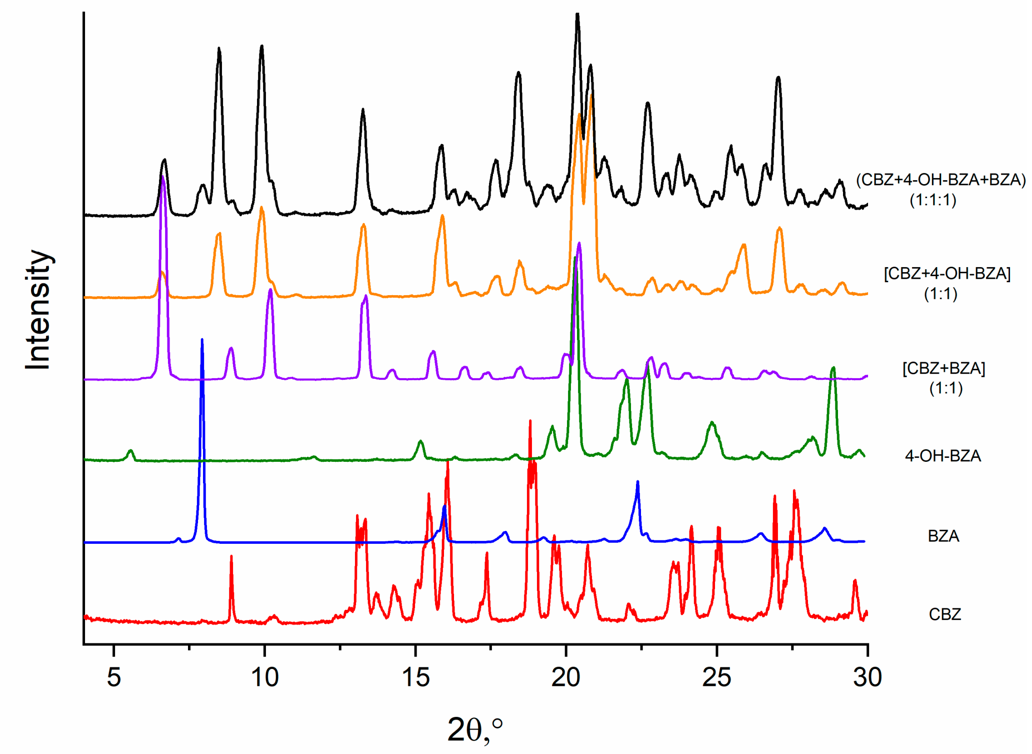

Figure S5. Comparison of the PXRD patterns: CBZ (red), BZA (blue), 4-OH-BZA (green), [CBZ+BZA] (1:1) (violet), [CBZ+4-OH-BZA] (1:1) (orange) and (CBZ+4-OH-BZA+BZA) (1:1:1) (black).

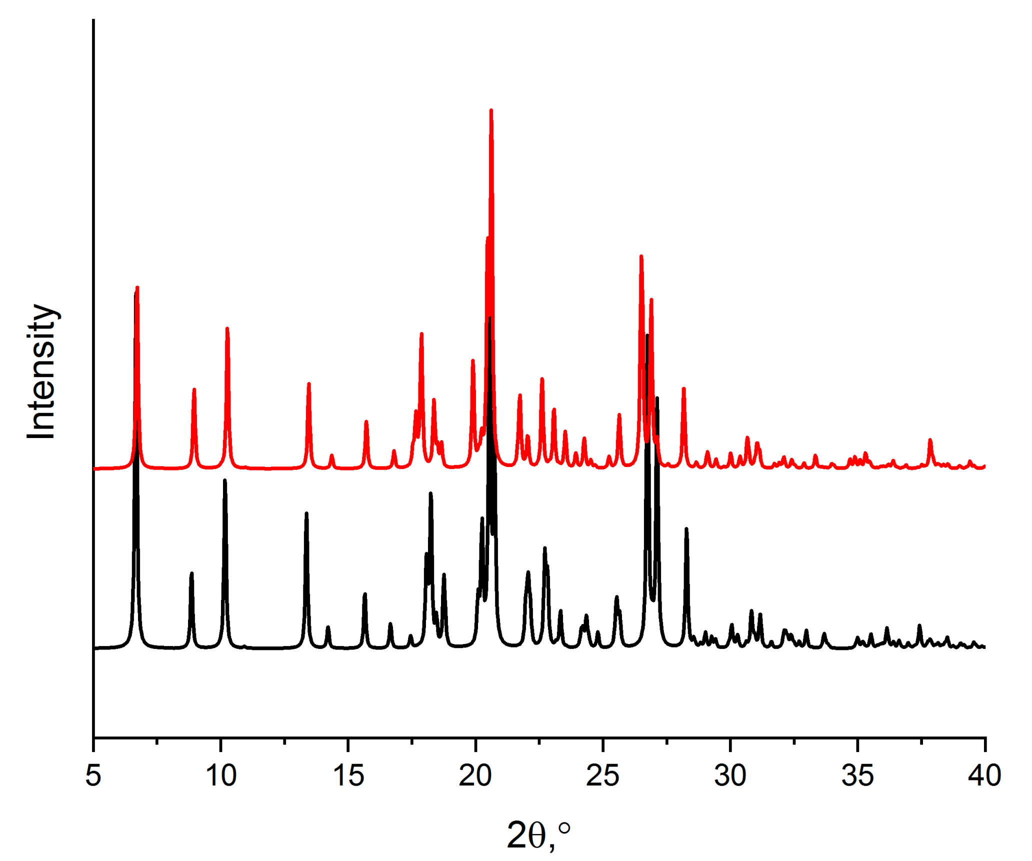

Figure S6. Comparison of the simulated PXRD patterns of [CBZ+BZA] (1:1) (black) and [CBZ+INAM] (1:1) form II (ref. LOFKIB01) (red).

References

1. Shayanfar, A.; Asadpour-Zeynali, K.; Jouyban, A. Solubility and dissolution rate of a carbamazepine–cinnamic acid cocrystal. *J. Mol. Liq.* **2013**, *178*, 171–176. https://doi.org/10.1016/j.molliq.2013.06.015.
2. Huang, D.; Chan, H.C.S.; Wu, Y.; Li, L.; Zhang, L.; Lv, Y.; Yang, X.; Zhou, Z. Phase solubility investigation and theoretical calculations on drug-drug cocrystals of carbamazepine with Emodin, Paeonol. *J. Mol. Liq.* **2021**, *329*, 115604. https://doi.org/10.1016/j.molliq.2021.115604.
3. Good, D.J.; Rodríguez-Hornedo, N. Solubility advantage of pharmaceutical cocrystals. *Cryst. Growth Des*. **2009**, *9*, 2252–2264. https://doi.org/10.1021/cg801039j.
4. Oliveira, M.A.; Peterson, M.L.; Davey, R.J. *Cryst. Growth Des.* **2011**, *11*, 449–457. https://doi.org/10.1021/cg101214m.
